# Electrolysis of Bacteria Based on Microfluidic Technology

**DOI:** 10.3390/mi14010144

**Published:** 2023-01-05

**Authors:** Jianqiu Zhao, Na Li, Xinyu Zhou, Zihan Yu, Mei Lan, Siyu Chen, Jiajia Miao, Yulai Li, Guiying Li, Fang Yang

**Affiliations:** Key Laboratory for Molecular Enzymology and Engineering of Ministry of Education, School of Life Sciences, Jilin University, Changchun 130012, China

**Keywords:** cell lysis, *E. coli*, microfluidic, AC electric field, electroporation

## Abstract

Cell lysis is a key step for studying the structure and function of proteins in cells and an important intermediate step in drug screening, cancer diagnosis, and genome analysis. The current cell lysis methods still suffer from limitations, such as the need for large instruments, a long and time-consuming process, a large sample volume, chemical reagent contamination, and their unsuitability for the small amount of bacteria lysis required for point-of-care testing (POCT) devices. Therefore, a fast, chemical-free, portable, and non-invasive device needs to be developed. In the present study, we designed an integrated microfluidic chip to achieve *E. coli* lysis by applying an alternating current (AC) electric field and investigated the effects of voltage, frequency, and flow rate on the lysis. The results showed that the lysis efficiency of the bacteria was increased with a higher voltage, lower frequency, and lower flow rate. When the voltage was at 10 V_p-p_, the lysis efficiency was close to 100%. The study provided a simple, rapid, reagent-free, and high-efficiency cleavage method for biology and biomedical applications involving bacteria lysis.

## 1. Introduction

Cell lysis is a basic step in biological research that uses an external force to break the cell membrane and release cellular contents, such as DNA, RNA, and proteins [1]. At present, physical, chemical, and biological methods are mainly used for cell lysis. However, there are still inevitable limitations to the traditional lysis methods, such as the high cost and the large damage to proteins by physical methods, the use of additional chemical reagents, and the difficulty of removing unrelated molecules and impurities. Biolysis methods are generally poorly applicable and are not suitable for large-scale extraction [2].

Microfluidic chips provide the advantages of miniaturization, integration, high throughput, low consumption, and rapid analysis, which can be meticulous and controllable in cell research conditions. Presently, the microfluidic cell lysis methods [3], including mechanical lysis, thermal lysis, laser lysis, acoustic lysis, electrolysis, chemical lysis, and enzymatic lysis, have successfully lysed cells [4,5] and bacteria [6,7]. Among these methods, microfluidic electrolysis of bacteria applies a strong electric field to the cell membrane so that the sample cells produce a strong transmembrane potential difference. It leads to pores on the cell membrane and causes unbalanced osmotic pressure to achieve the purpose of lysis. At the micro-scale, a higher electric field can be obtained at a lower voltage due to the small device size, so electrolysis can be widely used in small-scale systems such as microfluidic devices. The advantages of microfluidic electrolysis include high lysis efficiency and selectivity, with the cell membrane being broken while the organelle membrane is not affected [8]. The micron size of the channel is very suitable for single-cell isolation, detection, and analysis, so it can be directly integrated with highly sensitive detection equipment to realize a compact single-cell auto-analysis system with a low cost and high specificity [9,10]. Currently, this technology has achieved the lysis of red blood cells [11], HeLa cells [12], and *E. coli* [13]. However, these methods have shown to be quite complicated and costly to fabricate, many of them still depend on the use of high voltage and result in low lysis efficiency. Thus, a simple, efficient, and low-voltage electrical cell lysis method is highly desired.

In this paper, we present an electrical microfluidic bacteria lysis method and device, in which an interdigital electrode was employed. By applying low-voltage alternating current (AC) signals, highly effective bacterial lysis was successfully achieved. The parametric studies of electrical and fluidic conditions were conducted as well.

## 2. Materials and Methods

### 2.1. Theory Background

Microfluidic electrolysis applies an electric field to the cell membrane, and a strong transmembrane potential will be generated across the cell membrane. When the transmembrane potential is higher than the threshold potential, pores will be produced on the cell membrane and will cause an imbalance of osmotic pressure to lyse the cell [14]. In an AC electric field, the cell transmembrane potential difference can be expressed by Equation (1) [15,16]:

(1) $$\Delta V_{memb}=\frac{F\cdot a\cdot E\cdot cos\alpha }{{\left(1+{\left(\omega \tau \right)}^{2}\right)}^{\frac{1}{2}}}$$

where *F* is a factor determined by the shape of the cell (for spherical cells *F* = 1.5), *E* is the electric field strength, *a* is the cell radius, *α* is the electric field direction of the cell pole (*α* can be 0°–180°), *ω* is the frequency of the AC electric field, and *τ* is a coefficient determined by the cell membrane capacitance, cytoplasmic resistance, and cell suspension resistance.

**Figure 1:**
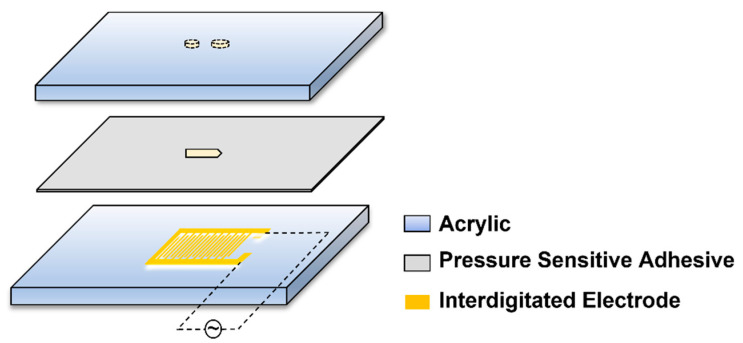
Schematic of the microfluidic chip.

It can be seen from the equation that the transmembrane potential generated on the bacterial cell membrane is related to the bacteria itself, the fluid properties, and the electric field strength and frequency. In the present study, we employed the interdigitated electrode, which has a 100 μm width with 100 μm spacing for each electrode in it, and the conductive layer was copper plated with gold (Figure 1). Due to the presence of multiple pairs of electrodes, the electric fields generated by each pair of electrodes were superimposed on each other, and the small electrode spacing highly increased the electric field strength. Therefore, the bacteria lysis was caused by a very strong electric field on a micro-scale.

### 2.2. Microfluidic Chip Fabrication and Integration

The microfluidic chip was composed of three layers (Figure 1), the upper layer consisted of a 1 mm-thick transparent acrylic sheet with inlet and outlet holes, the lower layer consisted of the electrode and the glass substrate, and the middle layer consisted of pressure-sensitive adhesive with the microchannel. The chip was fabricated by the lamination method developed previously in our lab [17]. The direction of the electric field was parallel to the channel. The width and length of the channel were 500–600 µm and 2 mm, the diameter of the inlet was 1 mm, and 1.8 mm for the outlet. The bacterial cells flowed into the inlet, and the bacterial lysate could be collected out of the outlet.

### 2.3. Experimental Setup

**Figure 2:**
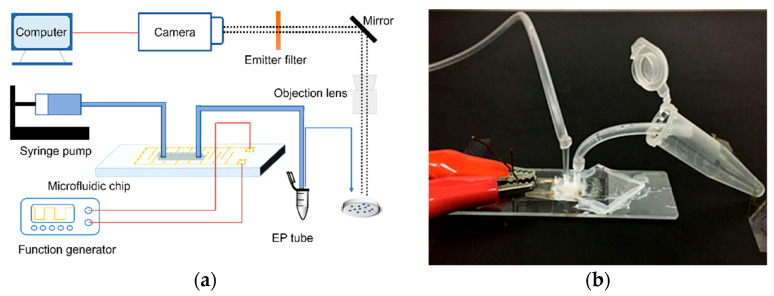
Schematic diagram of the experimental devices. (**a**) Experimental setup. (**b**) In the microfluidic chip, the left-side hose was used to inject *E. coli* dilution, and the right-side hose was used to drain the lysate into an EP tube for collection.

The experimental setup are shown in Figure 2. The *E. coli* suspension was injected into the chip channel by a syringe pump (Harvard, PHD2000, Holliston, MA, USA). The microfluidic chip, syringes, and hoses were sterilized with alcohol and rinsed with ultrapure water (UPW) before the experiments to ensure that no bacteria remained in the system. A function generator (Tektronix, AFG3102, Beaverton, OR, USA) was used as a power source to supply AC (with a phase shift of 180° between signals) with different voltages and frequencies to two electrodes on the chip. We changed the voltage in the range of 0–10 V_p-p_, the frequency of 1 kHz–50 MHz, and the flow rate of 0.5–10 μL/min. The lysate was collected in an EP tube, and then we counted the growth of colonies on LB solid culture plates. Since our chip was opaque, 10 μL of lysate from different lysis conditions were collected and observed under the microscope directly. A fluorescent microscope (Olympus-IX73, Tokyo, Japan) with a CCD camera (Olympus DP74, Tokyo, Japan) was used to visualize the results of bacterial lysis.

### 2.4. Sample Preparation

The bacteria strain was *E. coli* (JM109), and both the liquid and solid medium were LB medium. Cryopreserved *E. coli* was streaked onto LB solid medium and incubated for 18 h at 37 °C. A single colony was picked and inoculated in 5 mL of LB liquid medium, then placed in a constant temperature shaker at 37 °C and incubated at 180–200 rpm with shaking for 12 h until the OD 600 was approximately 1.0. Then, 1 mL of the bacterial solution was centrifuged at 130,000 rpm for 1 min, and the supernatant was decanted, then resuspended and diluted by UPW.

### 2.5. Statistical Analysis

The lysis efficiency was defined as Equation (2):

(2) $$\eta =\frac{n_{0}-n}{n_{0}}$$

where *n* was the number of colonies on culture plates in each 100 μL of lysate, *n*_0_ was the number of colonies from the original bacterial solution and *η* was the ratio of *n*_0_ − *n* to *n*_0_. Three experiments were repeated. Using Microsoft Excel (Microsoft Inc., Redmond, WA, USA) for image rendering, and data processing. The fitting curves were fitted logarithmically using Microsoft Excel, and they show the lysis efficiency of the bacteria under different conditions. Error bars indicated the standard deviation of measurements (*n* ≥ 3).

## 3. Results

### 3.1. Bacteria Lysis under Low AC Voltage

**Figure 3:**
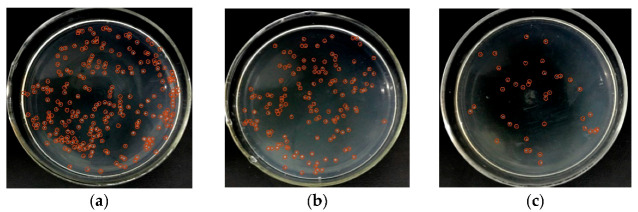
The activated interdigitated electrode enables efficient lysis of bacteria. (**a**) The result of bacteria without voltage. (**b**) When the voltage was 4 V_p-p_ and the frequency was 500 kHz, only a few *E. coli* were lysed. (**c**) The voltage was increased to 8 V_p-p_, and the number of colonies was reduced greatly.

To verify the ability of interdigital electroporation to lyse bacteria, we kept the frequency at 500 kHz and lysed *E. coli* at different voltages. As shown in Figure 3, when a low voltage (4 V_p-p_) was applied to the chip (Figure 3b), it could only partially lyse the *E. coli* compared to the control without voltage (Figure 3a). When the voltage was increased to 8 V_p-p_, the number of colonies on the plate coated with the lysate was greatly reduced (Figure 3c). The result confirmed that the interdigitated electrode could achieve efficient lysis of bacteria. When *E. coli* was located in an overlapping electric field, the electric field power forced its transmembrane potential and increase the lysis under osmotic pressure.

### 3.2. Voltage Effects on the Cell Lysis

**Figure 4:**
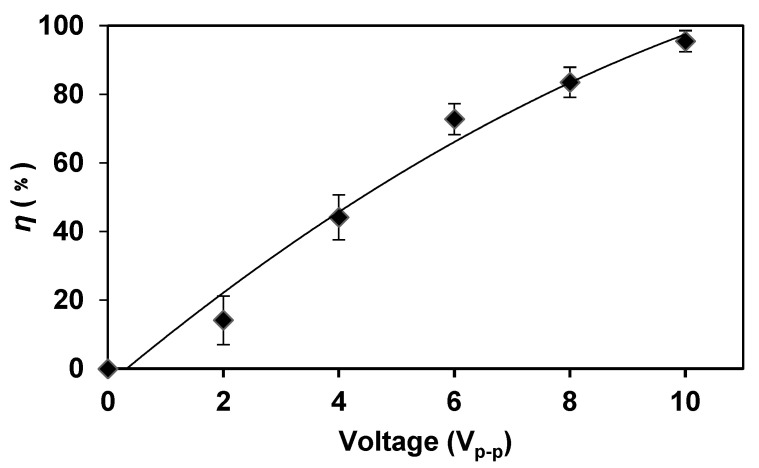
The higher voltage can better break down the bacteria to accelerate the lysis process compared with lower voltages. When the voltage was increased, the lysis efficiency was increased. The frequency was kept at 5 MHz and the flow rate was kept at 2 μL/min.

According to Equation (1), with the increase in the voltage level in the AC electric field, the electric field strength gradually increased, resulting in an increased cell transmembrane potential. Electroporation appeared and the cells became more susceptible to lyse. When the AC frequency and flow rate were kept constant, the lysis efficiency of *E. coli* increased along with the increase in voltage (Figure 4). When the voltage was 10 V_p-p_, the lysis efficiency was close to 100%, which indicated that higher voltages can better break down the bacteria to accelerate the lysis process compared with lower voltages.

We observed the *E. coli* lysis visually under the microscope. When the voltage was increased, the number of *E. coli* in the field of view decreased, which indicated that a higher voltage was more efficient for *E. coli* lysis. When the voltage was increased to 10 V_p-p_, almost no *E. coli* existed in the field of view, indicating that the lysis was quite complete.

### 3.3. Frequency Effects on the Cell Lysis

**Figure 5:**
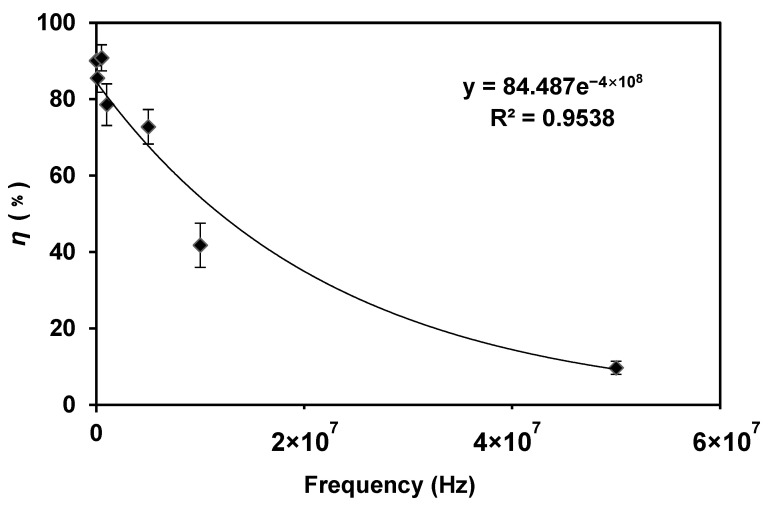
The lysis efficiency decreased when the frequency was increased. The voltage was 6 V_p-p_ and the flow rate was 2 μL/min. The frequency was changed in the range of 1 kHz–50 MHz.

According to Equation (1), the frequency is another factor that influences the transmembrane potential in an AC electric field; the cell transmembrane potential is inversely proportional to the electric field frequency. We kept the voltage of 6 V_p-p_ and the flow rate of 2 μL/min, the lysis results of different frequencies are shown in Figure 5.

When the frequency was in the range of 1–500 kHz, the efficiency of *E. coli* lysis was very high (about 90%). With the increase in frequency, the lysis efficiency decreased significantly. After the frequency reached 50 MHz, the bacteria could not be broken, indicating that the high-frequency condition was not suitable for bacteria lysis. This result is consistent with Equation (1), that is, with the decrease in frequency, the transmembrane potential increases gradually, and the lysis efficiency increases gradually.

To be more specific, according to Equation (1), (*ωτ*) is much less than 1 at low frequencies, i.e., (*ωτ*) *≪* 1. Therefore, the equation can be approximated at a low frequency as *ΔV* = (*F* · *a* · *cosα*) × *E*, the efficiency is predicted to be independent of frequency, as is shown in Figure 5. At high frequency (*ωτ*) ≫ 1.0, so Equation (1) can be approximate as

(3) $$\Delta V=\frac{F\cdot E\cdot a\cdot cos\alpha }{{\left(1+{\left(\omega \tau \right)}^{2}\right)}^{\frac{1}{2}}}=\frac{F\cdot E\cdot a\cdot cos\alpha }{\omega \tau }.$$

The above predicts that the efficiency is proportional to 1/*frequency* at high frequency. Thus,

(4) $$\Delta V=\frac{F\cdot E\cdot a\cdot cos\alpha \cdot /\tau }{\omega }.$$

At high frequencies, efficiency is inversely proportional to frequency, which agrees with our results in Figure 5, that a linear relationship was observed for frequencies above 1 kHz. However, more data might need to be acquired by an advanced function generator in our future study to further investigate this phenomenon.

### 3.4. Flow Rate Effects on the Cell Lysis

**Figure 6:**
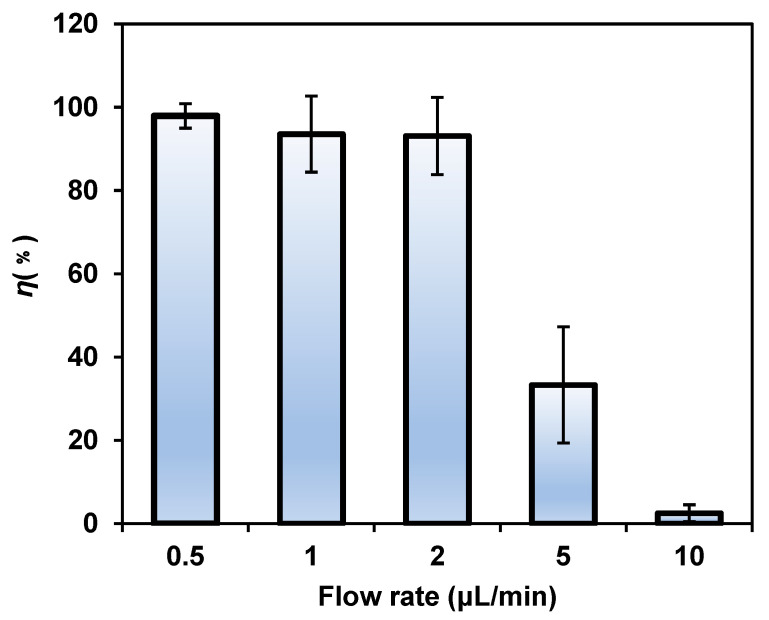
The lysis efficiency decreased significantly with the increase in the flow rate. The voltage was 8 V_p-p_ and the frequency was 1 kHz.

After investigating the effects of the electric field conditions on bacterial lysis, we found that the flow rate was also decisive for bacterial lysis efficiency (Figure 6). The results confirmed that when the flow rate was lower than 2 μL/min (3 s of processing time), there was no obvious difference in the lysis efficiency, which reached nearly 100%. When the flow rate was increased over 2 μL/min, the *E. coli* lysis efficiency showed a significant downward trend. When the flow rate reached 10 μL/min (0.6 s of processing time), the time that the bacteria spent in the AC field was relatively short, resulting in a large amount of recovered unlysed bacteria. The results showed that the flow rate can affect the lysis efficiency of bacteria by affecting the action time of the applied electric field on the bacteria. To improve the efficiency of the experiment and achieve high-throughput and high-efficiency bacteria lysis, the time required for bacteria lysis was minimized while ensuring lysis efficiency. Therefore, we considered 2 μL/min as the most suitable flow rate and 3 s as the shortest processing time for the present chip.

## 4. Conclusions

In this study, in order to realize the lysis of *E. coli* under low voltage conditions, we combined the interdigitated electrode with the microfluidic chip, which can achieve a strong electric field strength in a very small space, so that the *E. coli* cell membrane can generate a larger transmembrane potential and be cleaved more easily. Specifically, the lysis efficiency of *E. coli* at different voltages, frequencies, and flow rates were studied to achieve rapid, chemical-free, portable, and non-invasive bacterial lysis under low voltage conditions. The results showed that increasing the voltage and decreasing the frequency or flow rate can greatly improve the lysis efficiency of *E. coli*. Compared with conventional methods, this method is fast (3 s) and particularly suitable for small-volume sample lysis with no mechanical damage or chemical contamination [3]. Compared with other microfluidic-based electrolysis strategies [18,19], we achieved nearly 100% *E. coli* lysis efficiency at a lower voltage (10 V_p-p_ compared with thousands of volts) with an optimized frequency and flow rate. In addition, our microfluidic chip is simpler and less expensive to manufacture, enabling the efficient electrolysis of *E. coli*, which is suitable for basic research on trace bacteria.
